# High-accuracy prediction of mental health scores from English BERT embeddings trained on LLM-generated synthetic self-reports: a synthetic-only method development study

**DOI:** 10.3389/fdgth.2025.1694464

**Published:** 2026-01-08

**Authors:** Birger Moëll, Fredrik Sand Aronsson

**Affiliations:** 1 Division of Speech, Music and Hearing, School of Electrical Engineering and Computer Science, KTH Royal Institute of Technology, Stockholm, Sweden; 2 Division of Speech and Language Pathology, Department of Clinical Science, Intervention and Technology, Karolinska Institute, Stockholm, Sweden; 3 Theme Womens Health and Allied Health Professionals, Section of Speech and Language Pathology, Karolinska University Hospital, Stockholm, Sweden

**Keywords:** BERT, digital mental health, large language models, LSAS, natural language processing, PCL-5, PHQ-9, privacy-preserving evaluation

## Abstract

**Objective:**

To assess whether **synthetic-only** first-person clinical self-reports generated by a large language model (LLM) can support accurate prediction of standardized mental-health scores, enabling a privacy-preserving path for **method development and rapid prototyping** when real clinical text is unavailable.

**Methods:**

We prompted an LLM (Gemini 2.5; July 2025 snapshot) to produce **English-language** first-person narratives that are **paired with target scores** for three instruments—PHQ-9 (including suicidal ideation), LSAS, and PCL-5. **No real patients or clinical notes were used.** Narratives and labels were created synthetically and manually screened for coherence and label alignment. Each narrative was embedded using **bert-base-uncased** (mean-pooled 768-d vectors). We trained linear/regularized linear (Linear, Ridge, Lasso) and ensemble models (Random Forest, Gradient Boosting) for regression, and Logistic Regression/Random Forest for suicidal-ideation classification. Evaluation used 5-fold cross-validation (PHQ-9/SI) and 80/20 held-out splits (LSAS/PCL-5). Metrics: MSE, $R^{2}$, MAE; classification metrics are reported for SI.

**Results:**

Within the **synthetic distribution**, models fit the label–text signal strongly (e.g., PHQ-9 Ridge: MSE 4.41±0.56, $R^{2}\;0.92\pm 0.02$; LSAS Gradient Boosting test: MSE 75.00, $R^{2}\;0.95$; PCL-5 Ridge test: MSE 35.62, $R^{2}\;0.85$).

**Conclusions:**

LLM-generated self-reports encode a score-aligned signal that standard ML models can learn, indicating utility for **privacy-preserving, synthetic-only** prototyping. This is not a clinical tool: results **do not** imply generalization to real patient text. We clarify terminology (**synthetic text** vs. **real text**) and provide a roadmap for external validation, bias/fidelity assessment, and scope-limited deployment considerations before any clinical use.

## 1 Introduction

Mental health disorders represent a substantial and growing global health challenge, contributing significantly to disability and disease burden [1, 2]. Accurate, reliable, and timely assessment is paramount for effective diagnosis, treatment planning, and personalized care. Current methods, such as structured clinical interviews and self-report questionnaires like the Patient Health Questionnaire (PHQ-9), Liebowitz Social Anxiety Scale (LSAS) [3], and PTSD Checklist for DSM-5 (PCL-5) [4, 5], provide quantitative symptom severity measures based on established diagnostic frameworks [6, 7].

Despite their widespread use, these traditional methods have inherent limitations. Clinical interviews are time-consuming and require specialized expertise, limiting scalability. Self-reports rely on patient insight, memory, and willingness to disclose sensitive information, introducing potential biases. Furthermore, assessments often provide static snapshots, failing to capture dynamic symptom fluctuations over time.

Recent advancements in Natural Language Processing (NLP) and Machine Learning (ML), particularly Transformer-based architectures like BERT (Bidirectional Encoder Representations from Transformers) [8], offer powerful tools to augment these traditional methods [9]. Applying these techniques to patient-generated text holds promise for developing more objective and scalable methods for mental health monitoring. However, a critical impediment is the limited availability of large, high-quality, annotated clinical datasets. Patient data is inherently sensitive and protected by stringent privacy regulations globally (e.g., HIPAA, GDPR) [10], making data sharing ethically complex and logistically challenging. This data scarcity significantly slows the development of advanced NLP/ML models in mental health informatics.

Large Language Models (LLMs) like Gemini Team et al. [11] have demonstrated remarkable capabilities in generating fluent, human-like text across diverse domains [12, 13]. This presents a compelling opportunity to generate synthetic data—artificial data that mimics the statistical properties of real data without containing any actual patient information [14, 15]. Synthetic data is emerging as a potential strategy to address data scarcity and privacy concerns in sensitive domains like healthcare [14].

This study investigates the application of LLM-generated synthetic data within the context of NLP-based mental health assessment. Specifically, we evaluate the predictive performance of standard machine learning models trained on BERT embeddings derived exclusively from synthetic first-person clinical descriptions. The prediction targets are established quantitative scores for depression, social anxiety, and PTSD. The primary objective is to determine the extent to which models trained purely on synthetic data can accurately predict these scores within the synthetic dataset itself. Demonstrating high predictive accuracy in this controlled setting serves as an essential first step in evaluating the potential utility of this approach as a tool for research and model development where real data access is restricted.

### 1.1 Terminology and scope (synthetic vs. real text)

Throughout this manuscript, **synthetic text** refers to English first-person self-report narratives **generated by an LLM** based on specified target instrument scores. **Real text** refers to any authentic clinical or patient-authored text (e.g., clinical notes, genuine self-reports, or speech transcriptions) written by or about actual patients. This study is **synthetic-only**: we do not use any real patient data, and therefore no patient demographics exist for our datasets. The intended near-term use is **method development and benchmarking** (e.g., model selection, ablation, error analysis) when sharing or accessing real text is infeasible; at inference time in healthcare, the envisioned pipeline would consume **real** self-reports or notes, but that clinical generalization is future work and explicitly outside the scope here.

## 2 Literature review

The foundation of modern mental health assessment lies in instruments defined by the American Psychiatric Association’s DSM-5-TR [6]. These include structured clinical interviews and self-report scales like the PHQ-9 for depression, the Liebowitz Social Anxiety Scale (LSAS) [3], and the PCL-5 for PTSD [4]. While these instruments are standard, they are limited by their static, subjective, and resource-intensive nature.

The field of computational psychiatry has explored using NLP to create more scalable and objective assessment tools. Transformer-based models, such as BERT [8], have revolutionized NLP by learning deep, contextual language representations. These models have shown state-of-the-art performance on a variety of downstream tasks relevant to healthcare, including information extraction from clinical notes [9, 16]. However, progress is significantly impeded by the challenge of accessing large, high-quality clinical text datasets. Patient data is highly sensitive and protected by strict privacy regulations such as HIPAA in the US and GDPR in Europe, making data sharing for research logistically and ethically challenging [10]. This data scarcity is a well-documented bottleneck [17, 18].

Generative AI, specifically Large Language Models (LLMs), have demonstrated an ability to produce high-fidelity text [12, 13]. This has led to the emergence of synthetic data generation as a potential solution to data scarcity and privacy concerns in healthcare [14, 15]. Recent efforts have begun to leverage this potential, primarily using synthetic text for **data augmentation**—supplementing smaller, real datasets to improve model robustness and performance [19, 20]. This approach has shown promise in boosting performance on various clinical NLP tasks.

However, to our knowledge, no prior work has trained models **exclusively** on LLM-generated narratives to predict standard psychometric scores. Our study differs fundamentally from data augmentation strategies; by demonstrating the feasibility of training models that **fully forgo real text**, we explore a paradigm where the entire development and prototyping cycle can occur in a completely privacy-preserving sandbox. This sets the stage for evaluating the unique contribution of using a purely synthetic foundation for mental health assessment models, though the fidelity of this data and potential biases inherited from the LLM’s training remain critical considerations [21].

Prior healthcare studies have evaluated synthetic data for privacy-preserving development [14, 15], with mental-health–specific work increasingly exploring **augmentation** rather than **purely synthetic** training [e.g., creating additional narratives or paraphrases to bolster smaller real corpora; [19, 20]]. Reported benefits typically include improved robustness or class balance when real text is scarce; however, generalization relies on the presence of **some** real training data and careful blending strategies. By contrast, our study examines a **synthetic-only** regime to enable privacy-preserving prototyping when real text cannot be accessed or shared. To our knowledge, there are limited published resources that pair **free-text** narratives with gold LSAS or PCL-5 scores at scale; most assets provide item-level responses rather than open-form narratives. This underscores both the need for and the limits of synthetic-only experiments before any clinical evaluation.

## 3 Methods

### 3.1 Synthetic data generation

Three distinct datasets were generated using Gemini 2.5 PRO, an LLM accessed via its graphical user interface (GUI) in April of 2025. A multi-turn prompting strategy was employed, where initial prompts established the task context and format based on established clinical assessment forms for depression (PHQ-9, including suicidal ideation), social anxiety [LSAS; [3]], and PTSD [PCL-5; [4]], followed by iterative refinement. For instance, an initial prompt would request narratives grounded in specific item-level scores for a given instrument, and subsequent prompts would enforce a first-person perspective and consistent output structure. This conversational approach allowed the LLM to maintain context regarding the desired format and the clinical nature of the scales. Each generated narrative was manually reviewed for coherence and alignment between the text and its corresponding score. Table 1 provides representative examples of the generated narratives across different conditions and severity levels.

• **Depression (PHQ-9):** n=351 pairs (total score, description) and (suicidal ideation [0/1], description)
• **Social Anxiety (LSAS):** n=375 pairs (total score, description)
• **PTSD (PCL-5):** n=251 pairs (total score, description)

**Figure 1:**
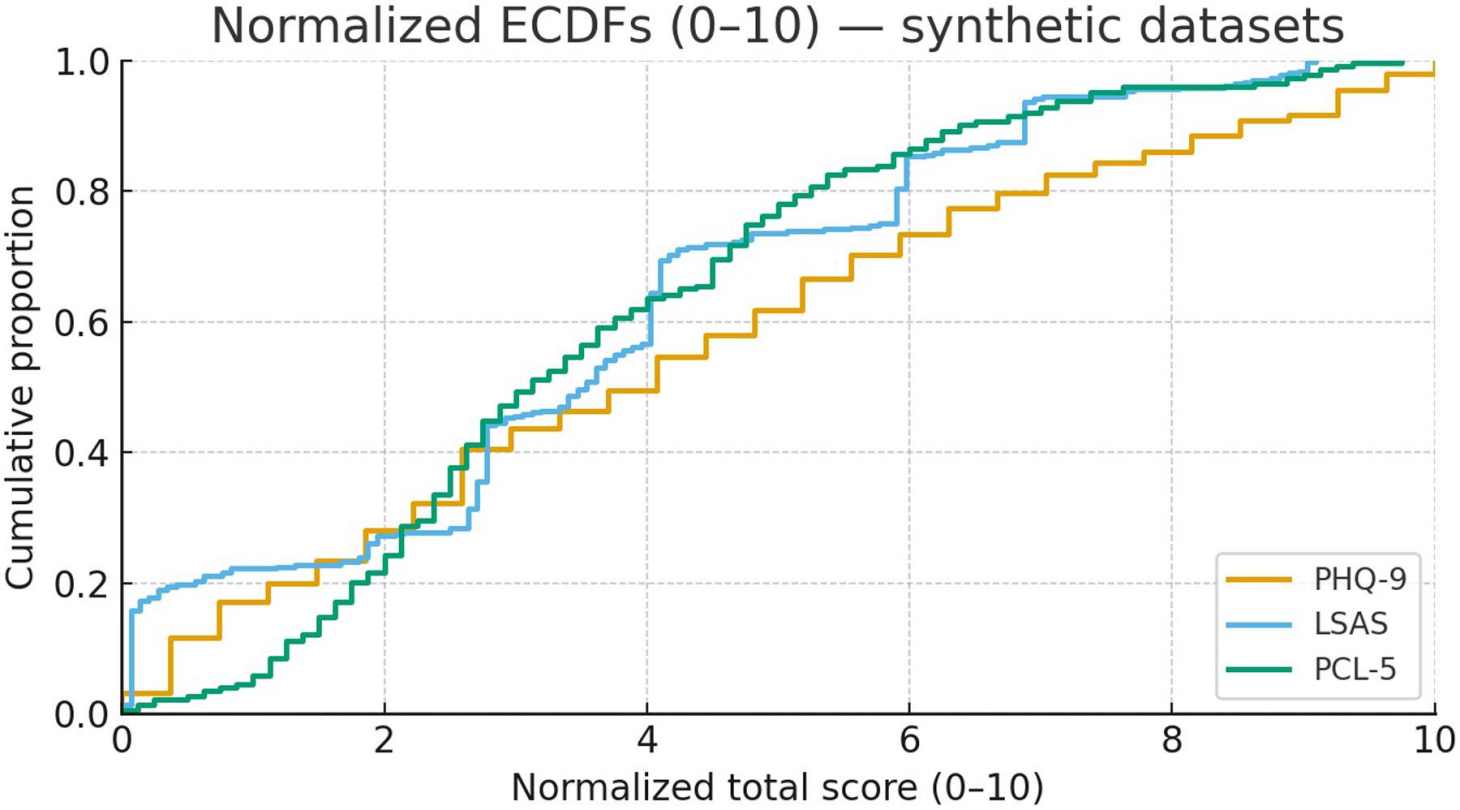
Normalized score distributions (0–10) for PHQ-9, LSAS, and PCL-5. Scores are scaled using theoretical ranges (PHQ-9: 0–27; LSAS: 0–144; PCL-5: 0–80).

Each dataset was randomly split into an 80% training set and a 20% held-out test set. The resulting score distributions for each instrument, normalized for comparison, are shown in Figure 1.

**Table 1:** Representative synthetic narratives at low/median/high scores across PHQ-9, LSAS, and PCL-5.

Domain	Score	Severity	SI	Example narrative (first-person)
Depression (PHQ-9)	1	Minimal/None	No	I’m feeling pretty good overall. Just maybe a day or two of lower energy, but it passes quickly and I still enjoy my usual activities. I’m sleeping fine and keeping up with work and family without trouble.
Depression (PHQ-9)	6	Mild depression	No	I just feel a bit off lately, kind of down. My motivation dips at times, but I’m still getting things done. I can focus when needed, though I occasionally withdraw from plans to rest and reset.
Depression (PHQ-9)	19	Moderately severe	Yes	Most days feel heavy and I’m exhausted even when I sleep. I’ve pulled away from people and struggle to see the point in things. Dark thoughts come up about not wanting to be here—when that happens I try to reach out or distract myself.
Social anxiety (LSAS)	26	Mild	–	I’m okay in familiar settings, but I get tense meeting new people or speaking up in small groups. My heart races a bit and I sometimes plan what to say ahead, but I usually manage and recover quickly afterwards.
Social anxiety (LSAS)	59	Moderate	–	I avoid speaking in meetings unless I must. Before social events I rehearse what I’ll say and worry others will judge me. I go but leave early to decompress. It takes energy to settle down after.
Social anxiety (LSAS)	92	Severe	–	I skip gatherings and presentations whenever possible. Even thinking about them triggers shaking and a tight chest. I cancel last-minute to avoid embarrassment and it’s starting to affect work and friendships.
PTSD (PCL-5)	9	Below threshold/Negative screen	–	I sometimes think about what happened, but it doesn’t interfere much. If I notice a reminder, I take a breath and ground myself. Sleep and concentration are mostly normal and I carry on with daily life.
PTSD (PCL-5)	33	Possible PTSD	–	Certain sounds or places take me back. I try to avoid them and feel on edge in crowds. Sleep isn’t great and I wake up tense. I can function day to day, but it’s effortful and I’m often drained.
PTSD (PCL-5)	56	Probable PTSD	–	Anything that reminds me of it hits hard—my heart races and I feel like I’m there again. I have frequent nightmares and avoid routes, people, and news that could trigger me. I’m jumpy, irritable, and worn out.

SI = Suicidal ideation (Yes/No). Examples are sampled from the synthetic datasets described in Section 3.1.

#### 3.1.1 Prompt exemplars

The prompts were iteratively refined in collaboration with the LLM during synthetic data generation. During this refinement, the goal was to make sure the LLM understood how to generate these texts and had the clinical understanding of the forms used such as LSAS.

##### 3.1.1.1 Seed Prompt (LSAS)

I want to make an experiment where I generate patient descriptions from assessment scores on the social phobia rating scale and then use LLMs to assess severity on that scale based on the written descriptions. Can you help me out with this.

##### 3.1.1.2 Follow-up Refinement.

Can you make five descriptions of scores on the LSAS with scores for all items and then write a description for each person.

##### 3.1.1.3 Style Constraint Exemplar.

I want the samples to be in first person format like the ones you provided earlier.

[High Severity Narrative]

Please start by making 10 samples.

##### 3.1.1.4 High-Severity Narrative (Illustrative LLM Output).

“My social anxiety controls my entire life. Almost every situation involving other people, whether it’s talking, being watched, performing, or just existing in public, causes intense fear and panic. My heart pounds, I feel short of breath, I tremble, and I’m completely convinced I’ll humiliate myself. Because of this, I avoid almost everything. I rarely leave my house except when absolutely necessary. I don’t attend social events, I avoid phone calls, I can’t speak up in any group setting, I can’t meet new people, and interacting with authority figures is terrifying. Even simple things like eating in front of others or returning items to a store feel impossible. I haven’t been able to pursue work or relationships properly. I feel trapped and isolated by the constant, overwhelming fear of judgment and scrutiny. It affects every single aspect of my daily functioning.”

### 3.2 Text preprocessing and embedding

The synthetic text underwent minimal preprocessing: conversion to lowercase and removal of whitespace. All narratives were generated and processed in English to match bert-base-uncased. We used the pre-trained bert-base-uncased model from the Hugging Face Transformers library [22] to convert the text into BERT embeddings. The process involved:

1. **Tokenization:** Breaking text into subword units.
2. **Padding & Truncation:** Uniformly sizing sequences to a maximum length of 512 tokens.
3. **Model Inference:** Passing tokenized inputs to the pre-trained BERT model.
4. **Mean Pooling:** Aggregating the last hidden layer’s outputs to create a single 768-dimensional vector embedding for each description.

### 3.3 Machine learning models

A suite of supervised learning models from the scikit-learn library [23] was used. For regression tasks (Depression, LSAS, PTSD scores), we evaluated:

• Linear Regression
• Ridge Regression (with L2 regularization)
• Lasso Regression (with L1 regularization, for PTSD)
• Random Forest Regressor
• Gradient Boosting Regressor (for LSAS)

For the binary classification task (suicidal ideation), we used:

• Logistic Regression
• Random Forest Classifier

A StandardScaler was used for linear models to standardize the embeddings. Hyperparameter tuning was performed using GridSearchCV with 5-fold cross-validation on the training set to optimize model performance.

### 3.4 Evaluation

Model performance was assessed using standard metrics:

• **Mean Squared Error (MSE):** $\text{MSE}=\frac{1}{n}\sum_{i=1}^{n}(y_{i}-{\hat{y}}_{i}{)}^{2}$. Lower is better.
• R**-squared ($R^{2}$):** $R^{2}=1-\frac{\sum_{i=1}^{n}(y_{i}-{\hat{y}}_{i}{)}^{2}}{\sum_{i=1}^{n}(y_{i}-\bar{y}{)}^{2}}$. Higher is better.
• **Mean Absolute Error (MAE):** $\text{MAE}=\frac{1}{n}\sum_{i=1}^{n}|y_{i}-{\hat{y}}_{i}|$. Lower is better.

Metrics were reported as the mean and standard deviation across cross-validation folds and on the final held-out test set.

## 4 System framework

The entire experimental pipeline can be summarized in the following steps, which represent the system framework used in this study.

1. **Synthetic Data Generation:** An LLM (Gemini 2.5) is prompted to generate first-person clinical descriptions paired with corresponding mental health scores.
2. **Text Preprocessing:** The generated text is cleaned and standardized (lowercase, no stemming).
3. **BERT Embedding:** The pre-processed text is converted into 768-dimensional contextual embeddings using a pre-trained bert-base-uncased model with mean pooling.
4. **Train-Test Split:** The dataset is partitioned into an 80% training set and a 20% held-out test set.
5. **Model Training & Hyperparameter Tuning:** Supervised machine learning models (e.g., Ridge Regression, Gradient Boosting) are trained on the embeddings from the training set. GridSearchCV with 5-fold cross-validation is used to find optimal hyperparameters.
6. **Performance Evaluation:** The trained models are used to make predictions on the held-out test set. MSE, $R^{2}$, and MAE are calculated to assess performance.

### 4.1 A note on clinical implementation

The LLM is only used **offline** to synthesize training/validation corpora for prototyping. The downstream prediction models (such as Ridge, Gradient Boosting) do not call the LLM at inference and operate on embeddings of input text. In a clinical setting, that input would be **real** self-reports or notes; this manuscript does not evaluate such generalization.

## 5 Results

The machine learning models, trained on BERT embeddings from the LLM-generated synthetic data, achieved high predictive performance across all three mental health conditions.

### 5.1 Depression and suicidal ideation

Table 2 summarizes the performance for depression score regression and suicidal ideation classification on the 5-fold cross-validation folds. Ridge Regression showed the best overall performance for the regression task, with low MSE and high $R^{2}$. For the binary classification of suicidal ideation, Logistic Regression demonstrated near-perfect accuracy.

**Table 2:** Performance on depression and suicidal ideation (5-fold CV).

Model	Task	Metric	Value (Mean ± Std. Dev.)
Ridge regression	Depression score (regression)	MSE	4.41±0.56
		$R^{2}$	0.92±0.02
		MAE	1.55±0.06
Logistic regression	Suicidal ideation (classification)	MSE	0.01±0.01
		$R^{2}$	0.94±0.05
		MAE	0.01±0.01
Random forest regressor	Depression score (regression)	MSE	5.38±0.84
		$R^{2}$	0.89±0.02
		MAE	1.75±0.09
Random forest classifier	Suicidal ideation (classification)	MSE	0.03±0.01
		$R^{2}$	0.86±0.03
		MAE	0.03±0.01

### 5.2 LSAS and PTSD score prediction

Table 3 presents the performance of the top-performing models on the held-out test sets for the LSAS and PCL-5 score prediction. Gradient Boosting Regressor achieved the lowest MSE and highest $R^{2}$ for the LSAS task, while Ridge Regression was the top performer for PCL-5. The standard Linear Regression model performed very poorly on the LSAS test set, demonstrating the necessity of regularization or robust ensemble methods for this high-dimensional feature space.

**Table 3:** Performance on LSAS and PCL-5 (held-out test set).

Condition	Best model	Test set MSE	Test set $R^{2}$
Social anxiety (LSAS)	Gradient boosting	75.00	0.95
	Ridge regression	76.94	0.95
	Random forest	105.25	0.93
	Linear regression	76,413	−50.63
PTSD (PCL-5)	Ridge regression	35.62	0.85
	Lasso regression	41.92	0.82
	Linear regression	52.51	0.78
	Random forest	59.53	0.75

### 5.3 Overall model performance

**Figure 2:**
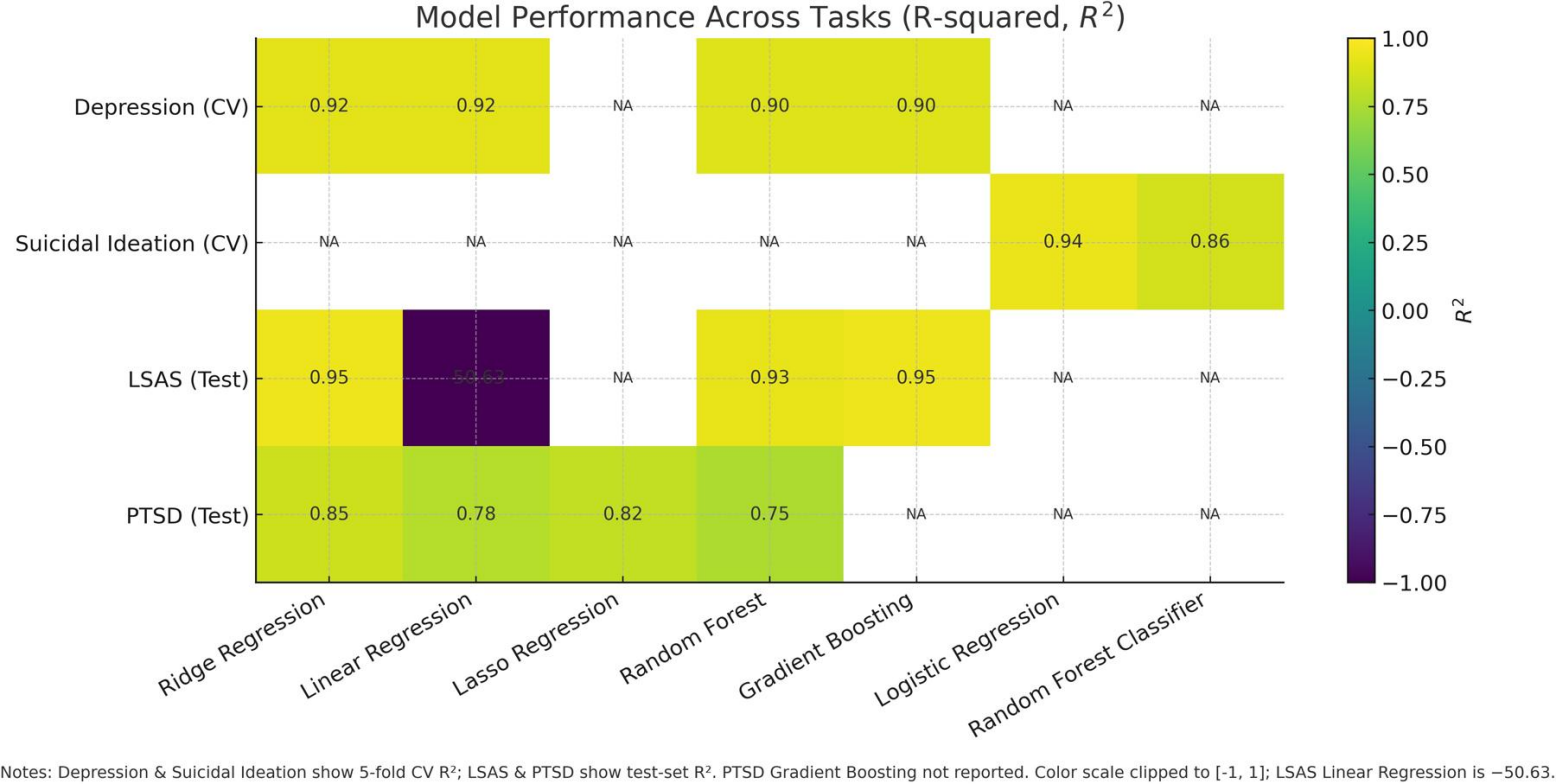
Model performance across tasks measured as $R^{2}$. Depression and Suicidal Ideation report 5-fold cross-validation; LSAS and PTSD report held-out test performance. Unreported model–task pairs are marked NA.

The results confirm that models trained on synthetic data can effectively capture the underlying relationships between text narratives and mental health scores within the synthetic domain. For an overview of model performance see Figure 2.

## 6 Discussion

This work demonstrates the feasibility of training mental health NLP models on BERT embeddings derived exclusively from LLM-generated synthetic data. We have shown that standard machine learning models can achieve high predictive accuracy for quantitative scores related to depression, social anxiety, and PTSD within this synthetic domain. This indicates that contemporary LLMs can generate text that contains a discernible, quantitative signal corresponding to symptom severity.

The strong and consistent performance of models like Ridge Regression and Gradient Boosting, as seen in Tables 2 and 3, highlights their effectiveness in handling the high-dimensional features produced by BERT embeddings. The exceptionally high $R^{2}$ values, however, require cautious interpretation. They are a measure of fit within the synthetic data distribution, which is likely more consistent and less noisy than real-world patient narratives. The synthetic data likely lacks the “messiness,” variability, and idiosyncrasies of genuine clinical language, which could make the learning task artificially easier.

Despite these caveats, a primary contribution of this study is highlighting LLM-based synthetic data generation as a valuable methodological tool. This approach offers a tangible pathway to circumvent long-standing hurdles related to patient privacy and the scarcity of annotated clinical datasets. It enables rapid prototyping, systematic exploration of different modeling architectures, and the potential creation of large, balanced datasets for specific research questions, thereby accelerating the research and development cycle for computational mental health tools.

### 6.1 Nuance and context loss in synthetic narratives

LLM-generated self-reports are typically coherent, well-structured, and symptom-explicit, which can exaggerate label–text alignment. By contrast, real psychiatric communication frequently includes (i) indirectness or denial of symptoms—especially around suicidal ideation (SI) [24], (ii) common co-morbidity (e.g., anxiety with depression, substance use) [25], (iii) fragmented or avoidant PTSD narratives and strong cue/context dependence [26–28], (iv) idiomatic, culture-specific, or multilingual expressions [29], and (v) variability in affective tone, narrative organization, education, dialect, and code-switching [29]. These factors weaken surface regularities and can diminish model performance when moving from synthetic to real text. Moreover, LLM style may impose format regularities (topic order, connective phrases) that function as **shortcut** cues for models [30]. Any deployment therefore requires audits for such artifacts and explicit robustness checks.

### 6.2 Position relative to augmentation/hybrid literature

Augmentation and hybrid designs (**real + synthetic**) often improve performance by enhancing data balance, coverage, and diversity when real text is limited [19, 20]. Our work complements this by showing that even in a **synthetic-only** setting, standard models can capture a strong signal of symptom severity from written narrative. This is useful for evaluating model families, embeddings, and hyperparameters without protected health information (PHI). For real-world deployment, we expect hybridization to be necessary, blending real text for fidelity with diversified synthetic variants for coverage, along with domain adaptation, calibration, and robust evaluation. For LSAS/PCL-5, we are not aware of any large-scale, public benchmarks that pair **free-text** narratives with gold scores, as most datasets provide item responses. Therefore, we present our contribution as a method-development scaffold that requires subsequent external validation on real text.

### 6.3 Limitations

The reliance on purely synthetic data is our study’s most significant limitation. There is no evidence presented here that models trained on this data will generalize effectively to authentic clinical text from real patients. The “domain gap” between synthetic and real data is likely substantial and remains unquantified.

### 6.4 Future work

Future research must rigorously focus on bridging the gap between synthetic prototyping and clinical reality. The most critical step is the external validation of these models on diverse, authentic clinical text to assess generalization. Simultaneously, comparative studies should be conducted to evaluate performance differences between models trained on synthetic-only, real-only, and hybrid datasets, allowing for the quantification of the specific value added by synthetic data. Further technical work is needed to investigate the impact of different LLMs and optimize prompting strategies for higher diversity and realism. Additionally, interpretability tools such as SHAP [31] and LIME [32] should be employed to audit for biases inherited from the LLM [18]. Finally, all future deployments must center on ethical considerations, ensuring that privacy-preserving methods do not inadvertently mask other forms of algorithmic bias [33].

### 6.5 Ethics and human subjects

This work used only LLM-generated synthetic text paired with synthetic labels; no human subjects, patient data, or protected health information were collected or analyzed. Institutional review was not required for these synthetic-only experiments.

## 7 Conclusion

This study provides compelling evidence that machine learning models using contextual BERT embeddings can achieve high predictive accuracy for depression, social anxiety, and PTSD assessment scores when trained and evaluated on synthetic clinical descriptions generated by a large language model. These findings highlight the significant potential of synthetic data generation as a valuable methodology to accelerate research and development in mental health informatics by mitigating persistent challenges related to clinical data access and patient privacy.

The impressive performance metrics observed within this purely synthetic environment must be interpreted with significant caution and should not be extrapolated directly to clinical practice. The crucial next phase of research involves extensive and rigorous validation of these models on diverse, real-world clinical datasets. This validation is an absolute prerequisite before any consideration can be given to translating these findings into tools intended for clinical use or decision support. Future work must concentrate on critically evaluating the fidelity, limitations, and potential biases of LLM-generated synthetic data, ensuring that the development and eventual deployment of NLP technologies in mental healthcare are conducted safely, effectively, ethically, and equitably.

In summary, LLM-generated synthetic data offers a promising stopgap solution that can enable research progress in mental health NLP without immediate access to sensitive clinical text. By demonstrating the high predictive performance in a controlled synthetic setting, we set the stage for subsequent studies to tackle the critical challenges of generalization and validation. If those challenges can be met, this approach could significantly accelerate innovation while respecting patient privacy – a win-win for clinical AI development.

## Data Availability

The raw data supporting the conclusions of this article will be made available by the authors, without undue reservation.
